# Dataset on the effects of CYB5D2 on the distribution of HeLa cervical cancer cell cycle

**DOI:** 10.1016/j.dib.2016.01.036

**Published:** 2016-01-28

**Authors:** Yanyun Xie, Yen Ting Shen, Anil Kapoor, Diane Ojo, Fengxiang Wei, Jason De Melo, Xiaozeng Lin, Nicholas Wong, Judy Yan, Lijian Tao, Pierre Major, Damu Tang

**Affiliations:** a Division of Nephrology, Department of Medicine, Xiangya Hospital, Central South University, Changsha, Hunan, PR China; b Division of Nephrology, Department of Medicine, McMaster University, Canada; c Father Sean O’Sullivan Research Institute, Canada; d The Hamilton Center for Kidney Research, St. Joseph’s Hospital, Canada; e Department of Surgery, McMaster University, Hamilton, Ontario, Canada; f The Genetics Laboratory, Institute of Women and Children’s Health, Longgang District, Shenzhen, Guangdong, PR China; g ZhunYi Medical University, Zhunyi, Guizhou, PR China; h Division of Medical Oncology, Department of Oncology, McMaster University, Hamilton, Ontario, Canada

## Abstract

We have recently reported that CYB5D2 plays a role in suppression of cervical cancer tumorigenesis, “CYB5D2 displays tumor suppression activities towards cervical cancer” [1]. We provide the accompany data here describing the effects of CYB5D2 overexpression and addition of recombinant CYB5D2 on HeLa cell cycle distribution. Furthermore, we will present the conditions used to specifically determine CYB5D2 expression in primary cervical and cervical cancer tissues using immunohistochemistry (IHC) and the patient cohort involved in assessing the CYB5D2 protein levels in primary cervical and cervical cancer tissues.

**Specifications Table**

**Table:** 

Subject area	Biology
More specific subject area	Cervical cancer tumorigenesis
Type of data	Figures, Table
How data was acquired	Western blot analysis using the Bio-Rad mini-gel apparatus; cell cycle determination using a flow cytometer (Bechman Coulter, Cytomics^TM^ FC500)
Data format	Filtered and analyzed
Experimental factors	Cells are serum-starved for 24 h, followed by stimulation with 10% of bovine fetal serum (FBS) to examine AKT and ERK activation
Experimental features	Cell cycle progression and protein expression
Data source location	Hamilton, Ontario, Canada
Data accessibility	Data is within this article

**Value of the data**

• CYB5D2׳s effects on HeLa cell cycle distribution could be considered when investigating a role of CYB5D2 in regulating cell proliferation in other cell types.
• The data on CYB5D2 in affecting ERK and AKT activation should be helpful in researching CYB5D2׳s role in regulating growth factor receptor signaling.
• The data is useful for future investigations of CYB5D2-mediated cellular processes.

## 1 Data

**Fig. 1:**
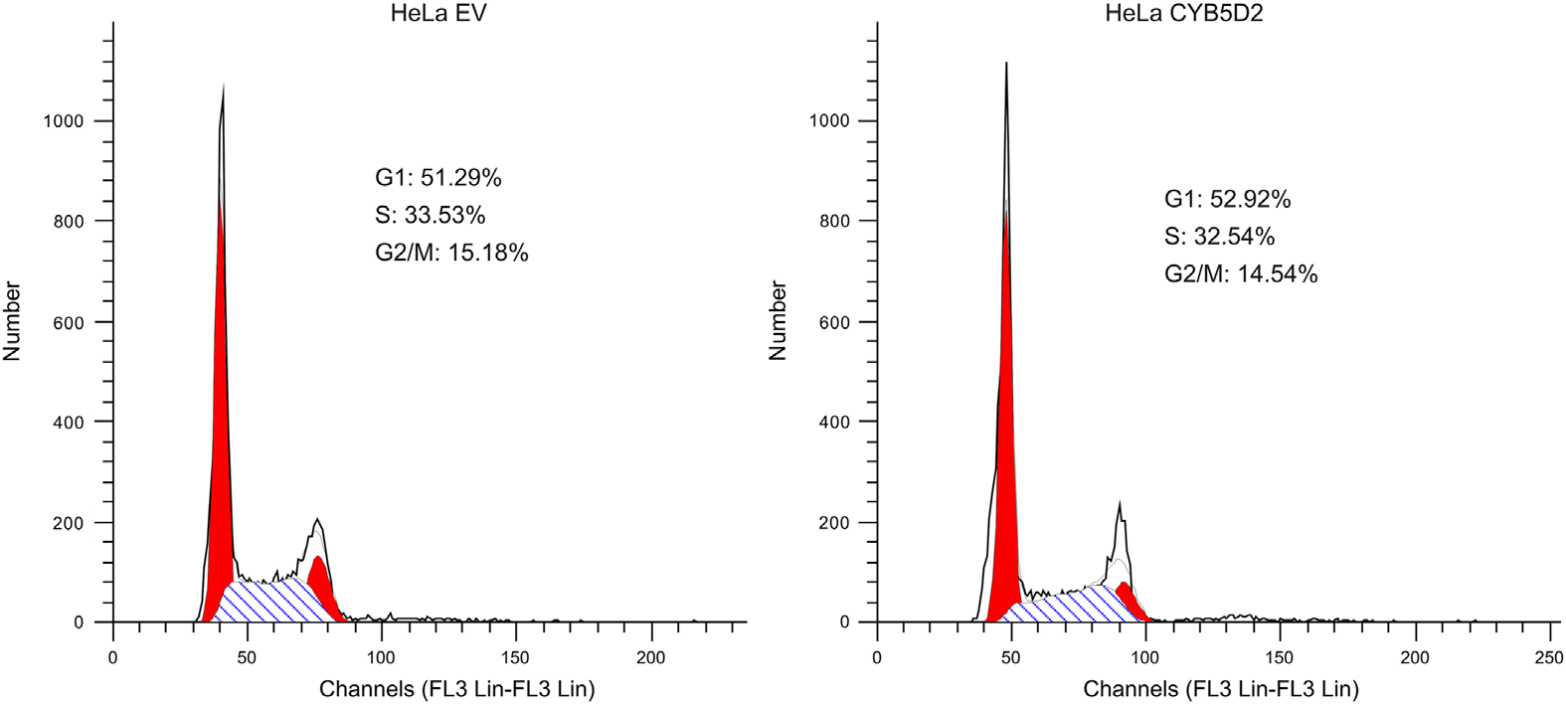
Examination of the effects of CYB5D2 overexpression on HeLa cell cycle distribution. HeLa EV and HeLa CYB5D2 cells were seeded in 60 mm plates, and cultured for 2 days. At density of approximately 80% confluency, cell cycle distributions were determined using a flow cytometer.

Fig. 1 examines the cell cycle distribution of HeLa cells stably expressing either an empty vector (HeLa EV) or CYB5D2 (HeLa CYB5D2).

**Fig. 2:**
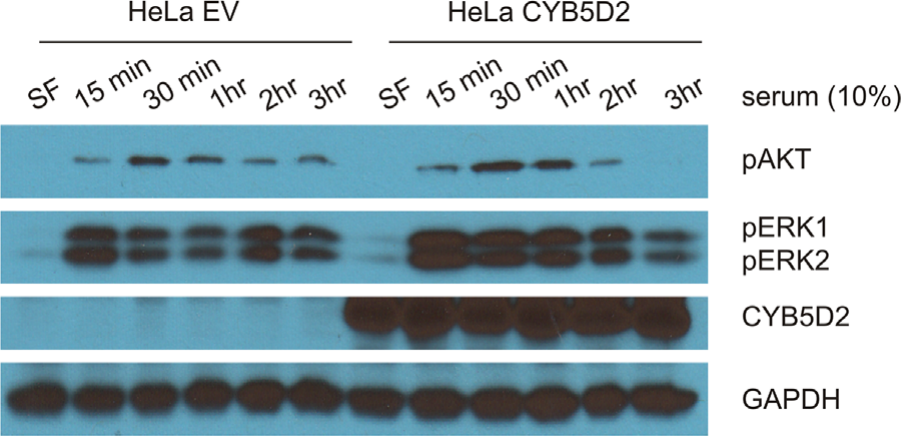
The impacts of ectopic expression of CYB5D2 on serum-induced activation of AKT and ERK kinases. HeLa EV and HeLa CYB5D2 cells at approximately 90% confluency were serum starved for 24 h, and stimulated with 10% of fetal bovine serum for the indicated periods, followed by western blot analysis for the phosphorylation of AKT at serine 473 (pAKT) and ERK at threonine 183 and tyrosine 185 (pERK1/2) as well as CYB5D2 and GAPDH. Experiments were performed twice; typical results from a single repeat are shown. SF: serum free.

Fig. 2 shows the status of AKT and ERK1/2 activation in HeLa EV and HeLa CYB5D2 cells. Activation of AKT and ERK1/2 was indirectly determined according to the specific phosphorylation events (see Fig. 2 legend for details).

**Fig. 3:**
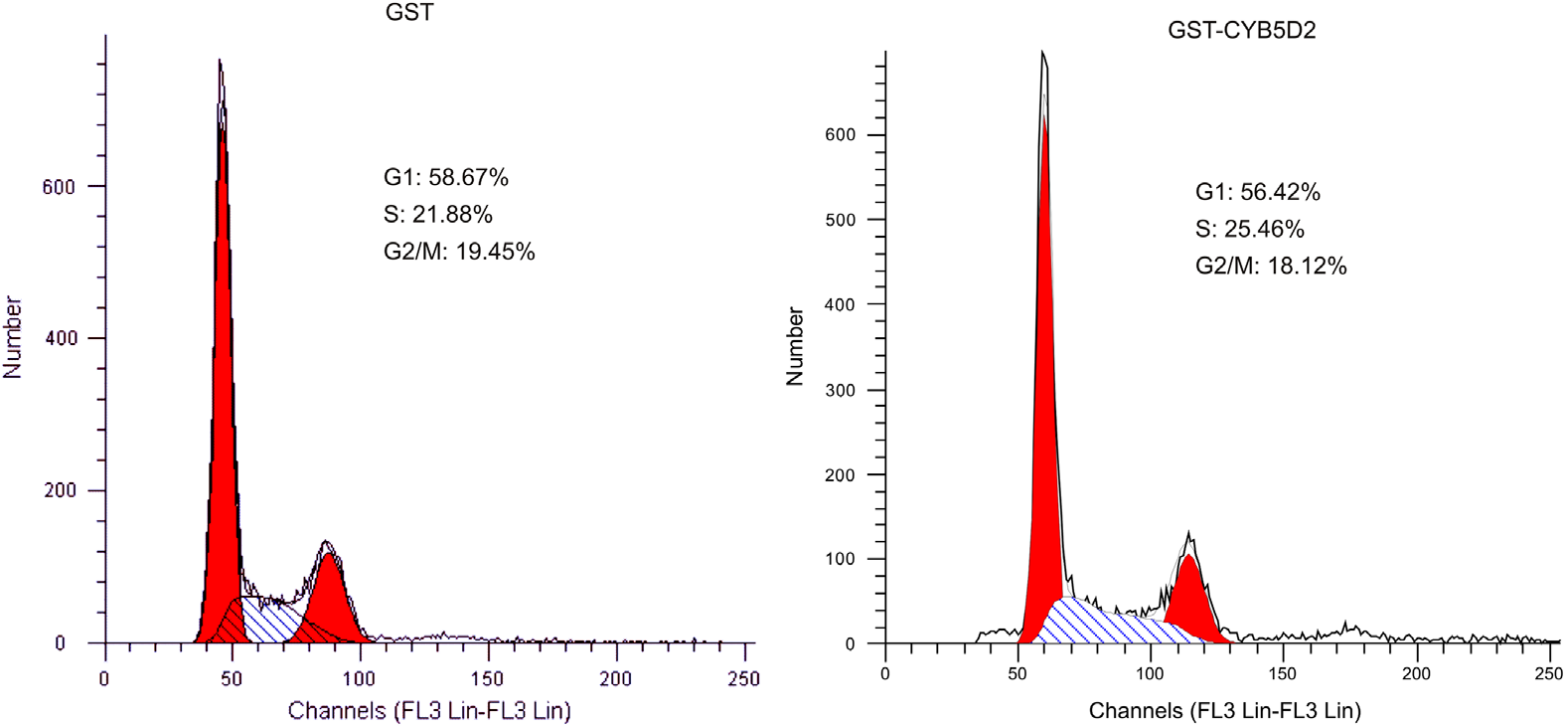
Determination of the effects of recombinant CYB5D2 on HeLa cell cycle distribution. GST and GST-CYB5D2 recombinant proteins were purified from *E. coli*. HeLa EV and HeLa CYB5D2 cells were incubated with GST and GST-CYB5D2 at 1 mg/ml for 24 h, followed by the determination of cell cycle distribution. Cell proliferation in the presence of either protein was clearly observed. Experiments were carried out twice; typical results from a single repeat are shown.

CYB5D2 can be a secretory protein [2], [3] that has been indicated to inhibit Neuro2a cell proliferation [2]. The cell cycle distribution of HeLa cells was determined in the presence of either GST or GST-CYB5D2 (Fig. 3).

**Fig. 4:**
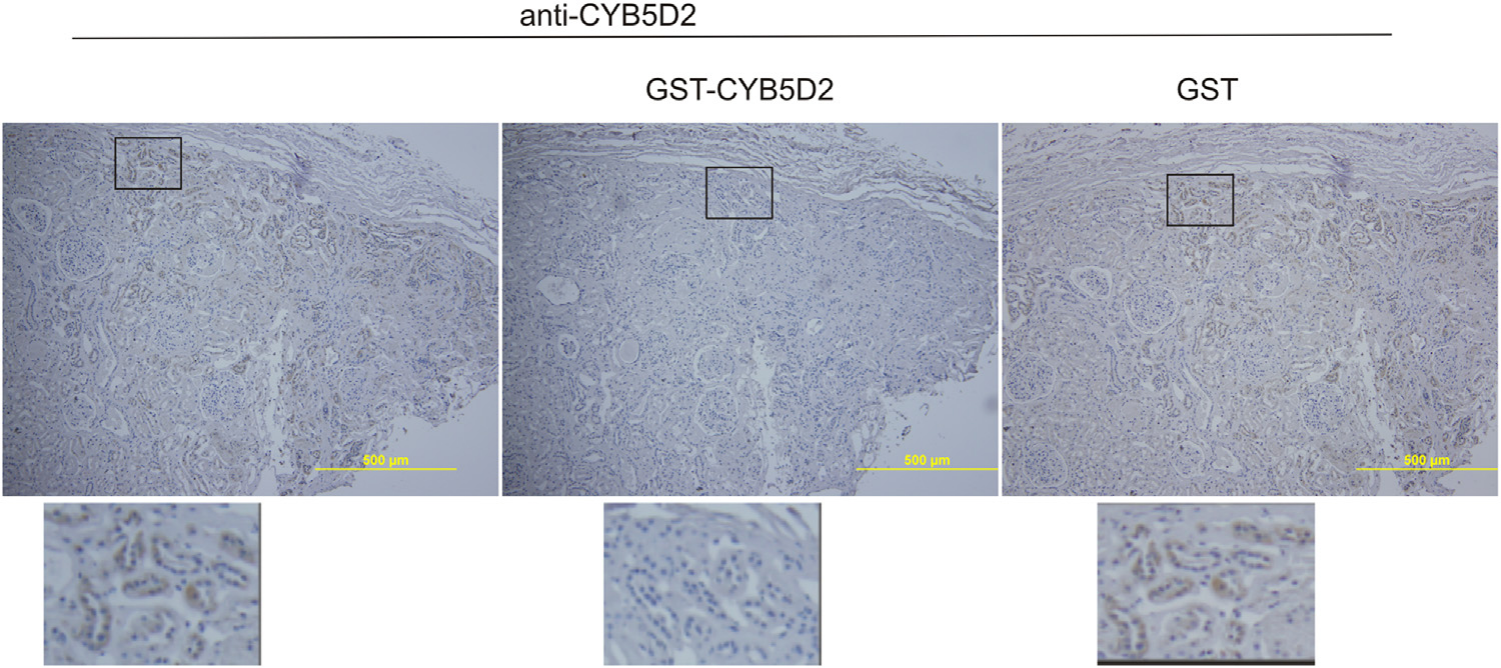
Anti-CYB5D2 antibody specifically recognizes CYB5D2. Normal human kidney tissue was immunohistochemistry (IHC) stained with anti-CYB5D2 antibody without and with addition of recombinant GST-CYB5D2 or GST. The indicated regions were enlarged 3 fold and presented underneath of the individual panels. Recombinant GST-CYB5D2 and GST were produced in *E. coli* BL21. The recombinant protein GST-CYB5D2 was generated by N-terminal fusion of the transmembrane domain deletion mutant of CYB5D2 to GST. Anti-CYB5D2 antibody was affinity-purified by using GST-CYB5D2 as previously described [4]. For the competition experiments, GST-CYB5D2 or GST at 1 mg/ml was pre-incubated for one hour on ice with anti-CYB5D2 antibody (1:250) before applying to human kidney tissues.

Fig. 4 shows recognition of the CYB5D2 protein in human kidney tissues by the anti-CYB5D2 antibody in the presence of GST or GST-CYB5D2 as a competitor.

Table 1 shows the tissues used to examine the CYB5D2 protein levels in normal cervical and cervical cancer tissues.

## 2 Experimental design, materials and methods

### 2.1 Experimental design and subjects

A tissue microarray slide was selected from US Biomax that contained 40 cervical squamous cell carcinoma and 20 normal cervical tissues (Table 1). HeLa cells stably expressing EV or CYB5D2 were recently constructed [1], [4].

### 2.2 Cell cycle distribution determination

Cell cycle distribution was determined by individualizing cells using 0.02% EDTA in PBS. Cells were stained with a propidium iodide (PI) solution (10 mM Tris pH7.5, 150 mM NaCl, 0.05 mg/ml PI, 0.1% sodium citrate, 0.2% Triton X-100, and 0.2 mg/ml DNase-free RNase A) overnight at 4 °C in dark.Cell cycle distribution was analyzed using a fluorescent automated cell sorting (FACS) (Bechman Coulter, Cytomics^TM^ FC500).

## Figures and Tables

**Table 1:** Patient׳s clinical information.

Patients	Pathological diagnosis	Age	Grade
1	Endocervical type adenocarcinoma	42	1
2	Endocervical type adenocarcinoma	42	1
3	Endometrioid adenocarcinoma with squamous metaplasia	48	1
4	Endometrioid adenocarcinoma with squamous metaplasia	48	1
5	Endocervical type adenocarcinoma	52	1–2
6	Endocervical type adenocarcinoma	52	1–2
7	Endometrioid adenocarcinoma	32	1–2
8	Endometrioid adenocarcinoma	32	1–2
9	Instestinal type adenocarcinoma	72	2
10	Instestinal type adenocarcinoma	72	2
11	Endocervical type adenocarcinoma	43	2
12	Endocervical type adenocarcinoma	43	2
13	Clear cell adenocarcinoma	40	–
14	Clear cell adenocarcinoma	40	–
15	Instestinal type adenocarcinoma	51	2
16	Instestinal type adenocarcinoma	51	2–3
17	Endocervical type adenocarcinoma	50	2–3
18	Endocervical type adenocarcinoma	50	2–3
19	Instestinal type adenocarcinoma	34	2
20	Instestinal type adenocarcinoma	34	2
21	Adenocarcinoma	44	3
22	Adenocarcinoma	44	3
23	Adenocarcinoma	52	3
24	Adenocarcinoma	52	3
25	Adenocarcinoma	59	3
26	Adenocarcinoma	59	3
27	Endometrioid adenocarcinoma	26	3
28	Endometrioid adenocarcinoma	26	3
29	Adenocarcinoma (fibrous tissue and blood vessel)	32	–
30	Adenocarcinoma (fibrous tissue and blood vessel)	32	–
31	Adenosquamous carcinoma	43	–
32	Adenosquamous carcinoma	43	–
33	Adenosquamous carcinoma	64	–
34	Adenosquamous carcinoma	64	–
35	Adenosquamous carcinoma	38	–
36	Adenosquamous carcinoma	38	–
37	Adenosquamous carcinoma	54	–
38	Adenosquamous carcinoma	54	–
39	Adenosquamous carcinoma	43	–
40	Adenosquamous carcinoma	43	–
41	Squamous cell carcinoma	53	2
42	Squamous cell carcinoma	53	2
43	Squamous cell carcinoma	27	2
44	Squamous cell carcinoma	27	2
45	Squamous cell carcinoma	68	2–3
46	Squamous cell carcinoma	68	2–3
47	Squamous cell carcinoma	37	3
48	Squamous cell carcinoma	37	3
49	Squamous cell carcinoma	43	3
50	Squamous cell carcinoma	43	3
51	Squamous cell carcinoma	69	2
52	Squamous cell carcinoma with necrosis	69	2
53	Squamous cell carcinoma (sparse)	48	2
54	Squamous cell carcinoma	48	2
55	Squamous cell carcinoma	36	3
56	Squamous cell carcinoma	36	3
57	Squamous cell carcinoma	63	2
58	Squamous cell carcinoma	63	2
59	Squamous cell carcinoma	47	2
60	Squamous cell carcinoma	47	1–2
61	Squamous cell carcinoma	40	2
62	Squamous cell carcinoma	40	2
63	Squamous cell carcinoma	76	2
64	Squamous cell carcinoma	76	2
65	Squamous cell carcinoma	38	3
66	Squamous cell carcinoma (fibrous tissue and blood vessel)	38	–
67	Squamous cell carcinoma	36	2–3
68	Squamous cell carcinoma	36	2–3
69	Squamous cell carcinoma	62	3
70	Squamous cell carcinoma	62	3
71	Squamous cell carcinoma	51	3
72	Squamous cell carcinoma	51	3
73	Squamous cell carcinoma	32	3
74	Squamous cell carcinoma	32	3
75	Squamous cell carcinoma	58	3
76	Squamous cell carcinoma	58	3
77	Squamous cell carcinoma	27	3
78	Squamous cell carcinoma	27	3
79	Squamous cell carcinoma	39	2
80	Squamous cell carcinoma	39	3
81	Cancer adjacent normal cervical tissue	45	–
82	Cancer adjacent normal cervical tissue	45	–
83	Cancer adjacent normal cervical canals tissue	62	–
84	Cancer adjacent normal cervical canals tissue	62	–
85	Cancer adjacent normal cervical canals tissue	50	–
86	Cancer adjacent normal cervical canals tissue	50	–
87	Cancer adjacent normal cervical tissue of No 13	40	–
88	Cancer adjacent normal cervical tissue of No 13	40	–
89	Cancer adjacent normal cervical tissue (fibrous tissue and blood vessel)	60	–
90	Cancer adjacent normal cervical tissue	60	–
91	Normal cervical tissue	18	–
92	Normal cervical tissue	18	–
93	Normal cervical tissue	15	–
94	Normal cervical tissue	15	–
95	Normal cervical tissue (fibrous tissue and blood vessel)	21	–
96	Normal cervical tissue (fibrous tissue and blood vessel)	21	–
97	Normal cervical tissue (with hyperplasia of glandular epithelium)	21	–
98	Normal cervical tissue (with hyperplasia of glandular epithelium)	21	–
99	Normal cervical tissue (fibrous tissue and blood vessel)	19	–
100	Normal cervical tissue (fibrous tissue and blood vessel)	19	–
