# Supplementary material for: Dataset on the effects of CYB5D2 on the distribution of HeLa cervical cancer cell cycle
Source: Data Brief. 2016 Jan 28;6:811–6. doi: 10.1016/j.dib.2016.01.036 (PMC4749936; doi:10.1016/j.dib.2016.01.036)
Supplement: Supplementary file 1 — Supplementary material [file mmc1.docx]

Dec 31, 2015

All authors declare no conflicts of interest for the manuscript, “Dataset on the effects of CYB5D2 on the distribution of HeLa cervical cancer cell cycle”.

**
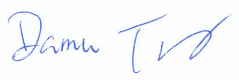
**

Damu Tang

On behalf of all authors

Associate Professor

Department of Medicine

McMaster University

Hamilton, ON

Canada

Tel: 905 522 1155, x35168

Fax: 905 521 6181

Email: damut@mcmaster.ca
